# Comparison of TiO_2_ and ZnO for Heterogeneous Photocatalytic Activation of the Peroxydisulfate Ion in Trimethoprim Degradation

**DOI:** 10.3390/ma16175920

**Published:** 2023-08-29

**Authors:** Máté Náfrádi, Tünde Alapi, Bence Veres, Luca Farkas, Gábor Bencsik, Csaba Janáky

**Affiliations:** 1 Department of Inorganic, Organic and Analytical Chemistry, University of Szeged, Dóm Square 7-8, H-6720 Szeged, Hungary; 7nmate7@gmail.com (M.N.); veres0629@gmail.com (B.V.); fluca@chem.u-szeged.hu (L.F.); 2 Department of Physical Chemistry and Materials Science, University of Szeged, Aradi Square 1, H-6720 Szeged, Hungary; bencsikg@chem.u-szeged.hu (G.B.); janaky@chem.u-szeged.hu (C.J.)

**Keywords:** advanced oxidation process, peroxydisulfate, matrix effect, domestic wastewater, pharmaceutical

## Abstract

The persulfate-based advanced oxidation process is a promising method for degrading organic pollutants. Herein, TiO_2_ and ZnO photocatalysts were combined with the peroxydisulfate ion (PDS) to enhance the efficiency. ZnO was significantly more efficient in PDS conversion and SO_4_^•−^ generation than TiO_2_. For ZnO, the PDS increased the transformation rate of the trimethoprim antibiotic from 1.58 × 10^−7^ M s^−1^ to 6.83 × 10^−7^ M s^−1^. However, in the case of TiO_2_, the moderated positive effect was manifested mainly in O_2_-free suspensions. The impact of dissolved O_2_ and trimethoprim on PDS transformation was also studied. The results reflected that the interaction of O_2_, PDS, and TRIM with the surface of the photocatalyst and their competition for photogenerated charges must be considered. The effect of radical scavengers confirmed that in addition to SO_4_^•−^, ^•^OH plays an essential role even in O_2_-free suspensions, and the contribution of SO_4_^•−^ to the transformation is much more significant for ZnO than for TiO_2_. The negative impact of biologically treated domestic wastewater as a matrix was manifested, most probably because of the radical scavenging capacity of Cl^−^ and HCO_3_^−^. Nevertheless, in the case of ZnO, the positive effect of PDS successfully overcompensates that, due to the efficient SO_4_^•−^ generation. Reusability tests were performed in Milli-Q water and biologically treated domestic wastewater, and only a slight decrease in the reactivity of ZnO photocatalysts was observed.

## 1. Introduction

Nowadays, environmental pollution, including water pollution, has received particular attention, and treating water pollutants has become an urgent task due to the biologically active and non-biodegradable organic contaminants, such as pharmaceuticals, which cannot be removed entirely using conventional water treatment methods. The global use of antibiotics and the increase of their concentration in wastewater is still growing [1,2,3]. During wastewater treatment, a significant part of the antibiotics and some of their metabolites can often remain in the effluent and reach surface waters, leading to the emergence of antibiotic-resistant bacterial strains, resulting in more than 70,000 deaths yearly worldwide [4]. Their emergence in wastewater and release to wastewater treatment plants can have significant effects, as they might act as reservoirs of antibiotic-resistant genes [5,6]. Therefore, effective elimination of these contaminants requires additive, tertiary water treatment methods and improvements in the existing technologies.

Advanced oxidation processes (AOPs) based on the in situ generation of strong oxidants have been developed to remove non-biodegradable organic pollutants. Most AOPs are based on the hydroxyl radical (^•^OH) generation, but in recent years methods based on the sulfate radical ion (SO_4_^•−^) have been gaining popularity [7,8]. The SO_4_^•−^ has a similar redox potential but a longer lifetime than ^•^OH [9,10]. Moreover, the easy activation of peroxymonosulfate (PMS) and peroxydisulfate (PDS) to produce SO_4_^•−^ makes this method favorable. The application of PDS is more advantageous due to its higher oxidation potential, stability, and lower production costs than PMS [11]. From PDS, the SO_4_^•−^ can be generated in situ by cleaving the O–O bond, which can be achieved by electron transfer [12,13] or by energy transfer through introducing energy in the form of photons [14,15] or heat [16]. In the case of different AOPs, the application of PDS is often mentioned as an alternative to the application of H_2_O_2_—the effectiveness of PDS-combined methods can usually exceed the H_2_O_2_-combined processes, partly due to the higher steady-state concentration of reactive species.

The excitation of the photocatalyst results in charge separation. The photogenerated electrons (e_CB_^−^) and holes (h_VB_^+^) can reduce and oxidize substrates adsorbed on the photocatalyst surface, respectively, or they can recombine with each other and disappear without leading to any chemical reaction. Consequently, an efficient e_CB_^−^ scavenger is required to prevent charge recombination and allow oxidation via h_VB_^+^. Most often, dissolved O_2_ reacts with e_CB_^−^. The formed O_2_^•−^ has a dual role: it can react with organic substances [17,18] or produce ^•^OH via a multistep reaction, especially in the case of TiO_2_ and ZnO photocatalysts [19].

In the case of heterogeneous photocatalysis, PDS can act as an e_CB_^−^ scavenger, like dissolved O_2._ The reaction with the e_CB_^−^ results in the formation of SO_4_^•−^; its oxidation potential and reactivity significantly exceed that of O_2_^•−^. Consequently, the combination of PDS with heterogeneous photocatalysis is expected to enhance the efficiency of the transformation and mineralization of organic substances, as has been confirmed by numerous publications [8,10,12]. PDS has been combined with various photocatalysts, including TiO_2_-based composites [20] and metal-oxide-based photocatalysts [21,22,23]. Its combination with metal-free graphitic carbon nitride (g-C_3_N_4_) [11] and visible light-active photocatalysts [23] is particularly beneficial.

Nevertheless, the combination of PDS and heterogeneous photocatalysis still raises many questions. The complexity of the process, such as the competition between PDS and O_2_, the simultaneous occurrence of radical and non-radical processes, and the interaction of various reactive species with each other, is challenging and needs further investigation [13,20,24,25,26,27].

The current research aims to compare the efficiency of commercial TiO_2_ and ZnO for removing an antibiotic, trimethoprim (TRIM), and to investigate the efficiency of combined TiO_2_/PDS and ZnO/PDS systems. The effects of reaction parameters, the formation of degradation products, the mineralization, and the reaction mechanisms were investigated and compared. Regarding practical applicability, it is essential to examine the matrix effect—for this, biologically purified domestic wastewater was used as a matrix.

## 2. Materials and Methods

### 2.1. Materials

The list of used chemicals and solvents can be found in Table S1. Trimethoprim (TRIM) was used as the target substance for photocatalytic test reactions. Air or N_2_ gas was applied to control the dissolved O_2_ concentration of the treated solution or suspension. To investigate the effect of peroxydisulfate ion (S_2_O_8_^2−^, PDS), Na_2_S_2_O_8_ solution was added to the suspension. Each material was used without further purification. Table S2 shows the data of the biologically treated domestic wastewater (from the water treatment plant, Szeged, Hungary) used as a matrix. Two commercially available photocatalysts, TiO_2_ Aeroxide^®^ P25 (Acros Organics; Geel, Antwerp, Belgium) and ZnO (d < 100 nm, Sigma Aldrich; St. Louise, MO, USA) photocatalysts were applied during the experiments. Section 2.4. contains the characterization of the photocatalysts.

### 2.2. Photoreactors and Experimental Parameters

**Figure 1:**
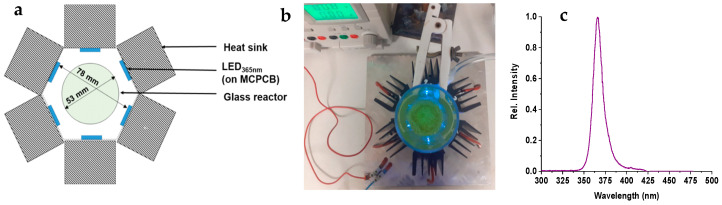
Schematic figure (**a**) and photo (**b**) of the applied photoreactor equipped with LEDs and the emission spectra of the LED (**c**).

The photoreactor was equipped with high-power UV-A LED (Vishay; Malvern, USA; VLMU3510-365-130; LED_365 nm_) emitting 355–380 nm light, with a UV-emission maximum of 365 nm (Figure 1). The 12 SMD diodes were soldered on metal-core printed circuit boards (Meodex; Narbonne, France) and fixed on six aluminum heat sinks (0.70 K W^−1^; Fischer Elektronik; Lüdenscheid, Germany); two LEDs were fixed on each heat sink. A laboratory power supply (Axiomet; Malmö, Sweden; AX-3005DBL-3, maximum output 5.0 A/30.0 V) provided and controlled the electrical power needed to operate the LEDs (P_el_^max^ = 21 W). The electrical power was optimized and fixed at 3.4 W. The glass reactor was placed in the center of the apparatus and surrounded by 12 LEDs (Figure 1).

In the case of each measurement, a 200 cm^3^ suspension was irradiated in a cylindrical borosilicate glass reactor. The dose of the photocatalyst was usually set to 1.0 g dm^−3^, except when the effect of the photocatalyst dosage was investigated. In that case, it was changed in the 0–1.5 g dm^−3^ range. The concentration of TRIM was 1.0 × 10^−4^ mol dm^−3^. The concentration of PDS was varied in the range of 0–5.0 mM, which was set by adding the appropriate volume of 0.5 M PDS stock solution in each case to the 200 mL suspension. The volume of the added stock solution at the highest PDS concentration (5.0 × 10^−3^ M) was 1.0 mL. The pH of the treated solutions and suspension was not adjusted. To investigate the role of radicals, experiments were performed in the presence of 1.0 × 10^−2^ M t- BuOH and 1.0 × 10^−2^ M MeOH.

The suspension was continuously stirred and bubbled with gas (N_2_ or synthetic air) during the treatments. The suspension containing photocatalysts and organic target substance was stirred and bubbled in the dark for 30 min to determine the amount of adsorbed target substance. The experiment was started by turning on the light source and adding the PDS solution to the suspension simultaneously. After sampling, 25 μL 0.3 M Na_2_S_2_O_3_ solution was added to the sample to decompose the remaining PDS. The photocatalyst samples were centrifuged immediately (Dragonlab; Beijing, China; 15,000 RPM) and filtered with syringe filters (FilterBio; Nantong, China; 0.22 µm, FilterBiO, PVDF-L) before further analysis.

### 2.3. Characterization of the Light Source

The emission spectra of the LED were measured using a two-channel fiber-optic CCD spectrometer (AvaSpec-FT2048; Avantes, The Netherlands) operated in the 180–880 nm wavelength range. The photon flux of the LEDs was measured using a potassium ferrioxalate actinometer [28]. 1.0 × 10^−2^ M ferrioxalate solutions were irradiated, and the solutions were bubbled with N_2_. The concentration of the released Fe^2+^ was determined after complexation with 1,10-phenanthroline. The concentration of the Fe^2+^-phenanthroline complex was measured using UV-Vis spectrophotometry (Agilent 8453; Santa Clara, CA, USA). The photon flux of the LED was 1.42 × 10^−5^ mol_photon_ s^−1^ dm^−3^, using 3.4 W electric power. The calculated average irradiance (flux density) reaching the reactor wall was approximately 5.6 mW cm^−2^.

### 2.4. Characterization of the Photocatalysts

The specific surface area TiO_2_ and ZnO was determined and found to be 64 and 13 m^2^ g^−1^, respectively. The average primary particle size for Aeroxide^®^ P25 TiO_2_ ranges from 10 to 50 nm, distributed mainly from 15 to 25 nm [29]. For ZnO, this value is 50–70 nm [30]. For TiO_2_, the XRD pattern agrees with the results reported in the literature; anatase is the dominant crystal phase in the anatase—rutile mixture [30,31]. The XRD pattern of ZnO confirmed its pure wurtzite phase [32].

**Figure 2:**
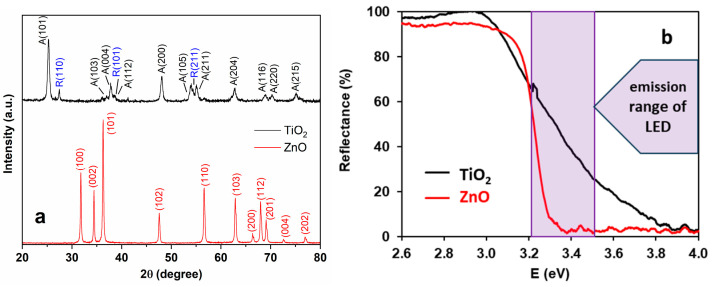
The XRD patterns and Miller indices (A: anatase; R: rutile) (**a**) and the diffuse reflectance spectra of TiO_2_ and ZnO (**b**).

Diffuse reflectance spectroscopy (DRS) was performed using an Ocean Optics USB4000 detector and Ocean Optics DH-2000 light source (Figure 2). The band gap energy values of the photocatalysts were evaluated by the Kubelka–Munk approach and the Tauc plot (Figure S1). The calculated band gaps were identical, 3.21 eV for TiO_2_ and ZnO (Figure S1).

### 2.5. Analytical Methods

The concentration of TRIM was measured by HPLC-DAD (Agilent 1100, Santa Clara, CA, USA; Gemini 3u C6-Phenyl 110A column; Phenomenex; Torrance, CA, USA). The eluent consisted of 10 *v*/*v*% methanol and 90 *v*/*v*% formate buffer (20 mM, pH = 3.7), the flow rate was 0.4 cm^3^ min^−1^, and the eluent temperature was 40 °C. The detection was performed at 275 nm, and the retention time of TRIM was 7.3 min.

The transformation efficiency of TRIM was characterized by the initial transformation rate (r_0_). This value was obtained from the slope of the first-order curve fitted to the points of the initial part of the kinetic curve of TRIM transformation (TRIM concentration (M) versus treatment time (s)). The R^2^ value of linear regression was above 0.95. Some experiments were repeated three times to check the reproducibility of the experimental results.

The TRIM degradation products were determined by HPLC-MS, with an Agilent LC/MSD VL mass spectrometer coupled to the HPLC system. The measurements were performed using an ESI ion source and a triple quadruple analyzer in positive mode (3000 V capillary voltage and 80 V fragmentor voltage). The drying gas flow rate was 8.0 dm^3^ min^−1^, and the temperature was 350 °C. The scanned mass range was between 50–1000 AMU. Solid-phase extraction (SPE) was used to remove dissolved inorganic ions before MS measurements. Phenomenex Strata-X, 33 μm cartridges, were used, and after conditioning (0.5 mL methanol and 0.5 mL water) 20 mL sample was loaded. Washing was performed using 1.0 mL water; then, the adsorbed samples were eluted using 0.75 mL methanol.

The sulfate ion (SO_4_^2−^) concentration was measured using ion chromatography (Shimadzu Prominence LC-20AD; Kyoto, Japan; Shodex NI-424 5U column). The eluent contained 2.3 × 10^−3^ mol dm^−3^ aminomethane, and the flow rate was 1.0 cm^3^ min^−1^.

Total organic carbon (TOC) concentration was determined using an Analytik Jena N/C 3100 analyzer (Analytik Jena; Jena, Germany). The adsorbable organic halogen (AOX) content was measured with a multi-X 2500 analyzer (Analytik Jena). The column method (APU-2) was used for the adsorption of 20 cm^3^ samples on 100 mg high-purity activated carbon.

## 3. Results

### 3.1. Effect of PDS and Dissolved O_2_ on the TRIM Transformation

Using 1.0 × 10^−4^ M TRIM and 1.0 g dm^−3^ photocatalyst dosage less than 5% of TRIM was adsorbed. The photolysis of TRIM was negligible, and no transformation was observed in the presence of a photocatalyst without irradiation. Using the highest PDS concentration (5.0 × 10^−3^ M), the slow TRIM transformation took place in the solution without photocatalyst under 365 nm radiation, most probably due to the photolysis of PDS (Figure S2), although its molar absorbance is negligible at this wavelength [7]. The competition with photocatalysts for photons in TiO_2_ and ZnO suspensions strongly reduces the possibility of direct PDS photolysis.

The effect of photocatalyst dosage was investigated in the 0–1.5 g dm^−3^ range. The transformation rate of TRIM did not change significantly above 0.5 g dm^−3^ (Figure S2) in the case of both photocatalysts. For further experiments, 1.0 g dm^−3^ TiO_2_ and ZnO concentration was used to ensure the complete absorption of the incoming photons by the photocatalyst and to minimize the possibility of direct photolysis of PDS. The TiO_2_ was slightly more efficient than ZnO in TRIM transformation (Figure S3), opposite to its less favorable optical properties. The better light absorption property of ZnO can be observed in the wavelength range emitted by LED_365 nm_ (355–380 nm) (Figure 2b); at 365 nm, TiO_2_ reflects approximately 40%, while ZnO practically fully absorbs the photons. The better efficiency of TiO_2_ in terms of TRIM transformation can be explained by the more efficient formation of ^•^OH, which was verified by Náfrádi [33], comparing TiO_2_ and ZnO in the case of coumarin transformation.

**Figure 3:**
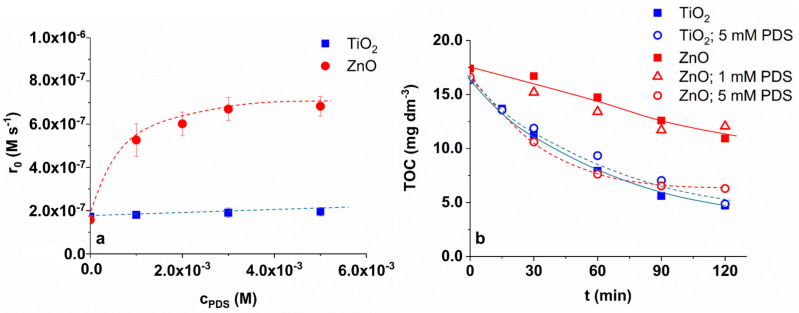
The initial transformation rate (r_0_) of TRIM as a function of PDS concentration (**a**) and the change of the total organic carbon (TOC) content using 1.0 × 10^−3^ M and 5.0 × 10^−3^ M PDS in aerated suspensions (**b**).

The effect of PDS concentration (0–5.0 × 10^−3^ M) was investigated in aerated suspensions containing 1.0 g dm^−3^ photocatalyst (Figure 3a). For TiO_2_, PDS just slightly enhances the transformation rate of TRIM (from 1.72 × 10^−7^ M s^−1^ to 1.95 × 10^−7^ M s^−1^); the effect is moderated, almost negligible. In contrast to TiO_2_, in the case of ZnO, the TRIM transformation rate increased significantly from 1.58 × 10^−7^ M s^−1^ to 6.83 × 10^−7^ M s^−1^ (Figure 3a). The effect varied according to a saturation curve; above 2.0 × 10^−3^ M, the efficiency cannot be significantly improved with further increase in PDS concentration. The positive effect is most likely caused by the formation of SO_4_^•−^ via electron transfer and its reaction with TRIM. The reactivity of TRIM towards SO_4_^•−^ is similar to ^•^OH (k_TRIM+•OH_ = 8.66 × 10^9^ M^−1^ s^−1^ [34,35]; k_TRIM+SO4•−_ = 3.88 × 10^9^ M^−1^ s^−1^ [35]). The enhancement of mineralization efficiency also could be observed. Due to the addition of 5.0 × 10^−3^ M PDS to ZnO suspension, the mineralization rate increased and reached the rate measured in the TiO_2_ suspension. (Figure 3b). For TiO_2_, PDS did not affect the mineralization rate, even at 5.0 × 10^−3^ M dosage.

The difference in the surface properties of ZnO and TiO_2_ partially explains their different behavior. Under the conditions applied (pH 6.8 and 7.3 for TiO_2_ and ZnO suspension, respectively), the surface charge of P25 TiO_2_ (pH_PZC_ = 5.2 and pH_IEP_ = 6.2 [36,37] is negative, while the surface charge of ZnO (pH_PZC_ = 8–9.4 [38,39,40,41] pH_IEP_ = 6.4–7.5 [42] is positive. For ZnO, a surface interaction was observed for several negative ions (carbonate, sulfate, and phosphate [43,44,45]), suggesting the possibility of a particular interaction with PDS, which can enhance the electron transfer from the surface of excited ZnO to PDS. However, for TiO_2_, no significant interaction was observed between the surface and inorganic anions [38].

Another essential difference between the photocatalysts could be in the ^•^OH formation process and the relative contribution of radical-based reactions and direct charge transfer to the transformation of organic and inorganic substances, which could be especially important for PDS activation. In the case of the anatase phase, ^•^OH formation is related primarily to the e_CB_^−^ initiated reduction of O_2_ and further transformation of O_2_^•−^ [43], while for ZnO, the similar contribution of h_VB_^+^ (^•^OH formation from H_2_O/OH^−^) and e_CB_^−^ initiated reactions have been suggested [46]. In the case of coumarin transformation, under the same experimental conditions, for ZnO, the direct charge transfer contributed to the transformation to a greater extent than for TiO_2_; for the latter, the ^•^OH-based transformation was the primary process [33]. Moreover, the higher electronic conductivity of ZnO can result in a more efficient charge accumulation on the surface compared to TiO_2_ [33,47,48]. Based on the above mentioned, the reduction of PDS by direct charge transfer on the ZnO surface may be more favorable than on the TiO_2_ surface, and its effect on the ^•^OH formation could be different for ZnO and TiO_2_.

### 3.2. Effect of Dissolved O_2_ on TRIM Transformation

**Figure 4:**
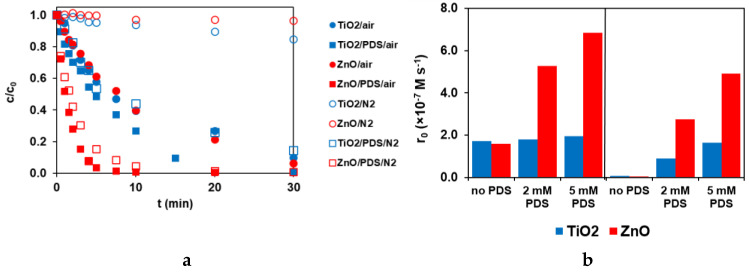
The effect of dissolved O_2_ and PDS on the transformation of TRIM in the case of ZnO and TiO_2_ in aerated and O_2_-free (N_2_) suspensions. (**a**: kinetic curves, c_PDS_ = 5.0 × 10^−3^ M; **b**: initial transformation rates).

The fast recombination of photogenerated charges occurs without an effective e_CB_^−^ scavenger. The dissolved O_2_ usually plays this role. The effect of O_2_ was investigated for both catalysts in aerated and O_2_-free suspensions, with and without PDS (Figure 4). Without PDS and dissolved O_2_, the transformation of TRIM is negligible in both suspensions. However, in the presence of PDS, the transformation occurs even in O_2_-free suspensions (Figure 4). PDS greatly enhanced the transformation rate in both O_2_-free and aerated ZnO suspensions (Figure 4). While in the case of TiO_2_, the effect of PDS depended on the presence of dissolved O_2_, it was very slight in the case of aerated but remarkable in the O_2_-free suspension (Figure 4). The co-presence of PDS and O_2_ and the complexity of their roles can explain the difference between TiO_2_ and ZnO.

The PDS is an excellent e_CB_^−^ scavenger that efficiently prevents the recombination of photogenerated charges (e_CB_^−^ and h_VB_^+^), enabling the reduction and oxidation of substrates on the surface of photocatalysts. The reaction between PDS and e_CB_^−^ results in the facile formation of highly oxidizing SO_4_^•−^, which opens a new pathway for the transformation of TRIM, with a similar rate to ^•^OH [35]. The simultaneous presence of PDS and O_2_ can affect the SO_4_^•−^ and O_2_^•−^ formation rate via competition for e_CB_^−^, and consequently, the transformation rate of organic compounds. The result of the competition also depends on the surface properties of the photocatalysts.

The role of O_2_^•−^ is not manifested in a direct reaction with TRIM [34] but in its contribution to ^•^OH formation. For TiO_2_ and ZnO, ^•^OH formation partly occurs via the further transformation of O_2_^•−^ and H_2_O_2_ [49]. The SO_4_^•−^ also can participate in pH-dependent reactions to produce ^•^OH [50,51], but ^•^OH formation in this way can only become significant above pH 9:

SO_4_^•−^ + H_2_O → H^+^ + SO_4_^2−^ + ^•^OH  k = 460 s^−1^ (1)

SO_4_^•−^ + OH^−^ → SO_4_^2−^ + ^•^OH  k = 6.5 × 10^7^  M^−1^ s^−1^ (2)

Nevertheless, the PDS as an e_CB_^−^ scavenger can enhance the ^•^OH formation via h_VB_^+^-driven processes. Thus, in PDS containing TiO_2_ and ZnO suspensions, both SO_4_^•−^ and ^•^OH can participate in the degradation processes and must be considered a decisive reactive species, even in an O_2_-free suspension. Moreover, the increased importance of the direct charge transfer (the reaction between TRIM and photogenerated h_VB_^+^) can be reasonably assumed in the presence of a potent electron scavenger, such as PDS.

The peroxyl radicals (R-OO^•^) produced during the transformation of organic compounds play an important role in their transformations and mineralization—from this point of view, the role of O_2_ is unique. For ZnO, the slight positive effect of dissolved O_2_ on the transformation of TRIM in the presence of PDS is presumably due to the organic peroxyl radicals, which opens a new path for transformations and simultaneously hinders the backward reactions of carbon-centered radicals. It should also be noted that the reaction between SO_4_^•−^ and ^•^OH results in O_2_ [52]:

SO_4_^•−^ + ^•^OH → H^+^ + SO_4_^2−^ + 0.5 O_2_   k = 9.5 × 10^9^   M^−1^ s^−1^ (3)

Consequently, O_2_-mediated transformation cannot be excluded completely, even in suspension bubbled with N_2_. 

### 3.3. Effect of Dissolved O_2_ and Trimethoprim on PDS Transformation 

**Figure 5:**
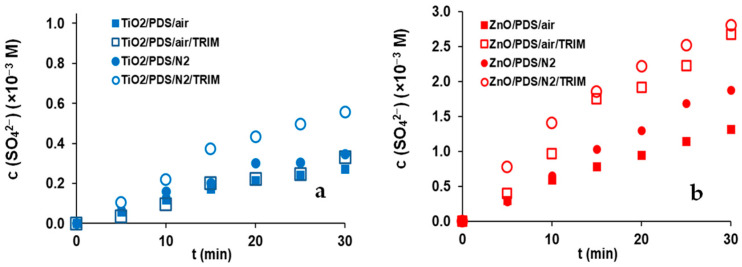
The effect of dissolved O_2_ and TRIM (1.0 × 10^−4^ M) on SO_4_^2−^ concentration (c_0_(PDS) = 2.0 × 10^−3^ M) in the case of TiO_2_ (**a**) and ZnO (**b**), in aerated and O_2_-free (N_2_) suspensions.

Most publications have focused primarily on the transformation of organic compounds and have rarely investigated the PDS transformation. The PDS (2.0 × 10^−3^ M) transformation was followed by the measurement of SO_4_^2−^ concentration. Experiments were conducted in aerated and O_2_-free suspensions with and without TRIM (Figure 5).

The SO_4_^2−^ formation is significantly faster in ZnO suspension than in TiO_2_ suspension (Figure 5, Table S3), especially in the presence of TRIM which is consistent with the effect of PDS on TRIM transformation (Figure 3) and proves that the PDS transformation is much more favored for ZnO than for TiO_2_. Without TRIM, 9% and 12% of PDS were transformed for TiO_2_ and 52% and 69% for ZnO in aerated and O_2_-free suspension over 60 min treatment, confirming the competition for e_CB_^−^ between O_2_ and PDS. This competition was much more pronounced in the case of ZnO. In addition to dissolved O_2_, TRIM also influenced the transformation of PDS. In an O_2_-free TiO_2_ suspension, the SO_4_^2−^ concentration increased by 70% due to the presence of TRIM; however, this effect was negligible in aerated suspension. In ZnO suspension, the positive impact of TRIM was manifested both in O_2_-free and O_2_-containing suspensions but still, the extent of the effect depended on the presence of O_2_: in an O_2_-free suspension 47% increase was achieved, while in an aerated suspension, the SO_4_^2−^ concentration was doubled.

In the case of TiO_2_-based composite photocatalysts, a positive effect of PDS and PMS on the transformation of organic substances was reported and interpreted by the dual roles of PMS as a surface complexing ligand [53] and a radical precursor. The oxidizing capacity of TiO_2_/PMS varied depending on the substrate type [53]. While in the case of dye transformation, photosensitization has an important role [54]. Based on these results, we cannot determine the processes taking place on the surface, but the results do prove that not only the competition between PDS and O_2_ but also the interaction of TRIM and PDS with the catalyst surface can affect the transformation. Moreover, depending on the presence of O_2_ and photocatalyst properties, different products can form from TRIM, and some of them, such as quinones and phenols, can promote the transformation of PDS [55].

All this suggests that during the combination of PDS and heterogeneous photocatalysis, it may become necessary to consider the interaction of individual materials with the photocatalyst surface or with each other to interpret the processes taking place on the surface and their effect on overall efficiency.

### 3.4. Effect of Radical Scavengers 

The contribution of ^•^OH and SO_4_^•−^ to the TRIM transformation was studied by competition kinetics, using radical scavengers. Tert-butanol (t-BuOH) was used as ^•^OH scavenger (k_t-BuOH+•OH_ = 6.0 × 10^8^ M^−1^ s^−1^ [56]; k_t-BuOH+SO4•−_ = 8.0 × 10^5^ M^−1^ s^−1^ [57]) and methanol (MeOH) was applied as ^•^OH (k_MeOH+•OH_ = 9.7 × 10^8^ M^−1^ s^−1^ [56,57] and SO_4_^•−^ (k_MeOH+SO4•−_ = 2.0 × 10^7^ M^−1^ s^−1^ [57,58]) scavenger. Comparing their effect, the contribution of ^•^OH and SO_4_^•−^ to the transformation of TRIM (k_TRIM+•OH_ = 8.66 × 10^9^ M^−1^ s^−1^ [34,35]; k_TRIM+SO4•−_ = 3.88 × 10^5^ M^−1^ s^−1^ [35]) can be estimated. Experiments were performed in the presence of 1.0 × 10^−2^ M t-BuOH and MeOH. Both alcohols theoretically scavenge ~90% of ^•^OH (RSC ≈ 0.91 and MeOH reacts with ~20% of SO_4_^•−^.

**Figure 6:**
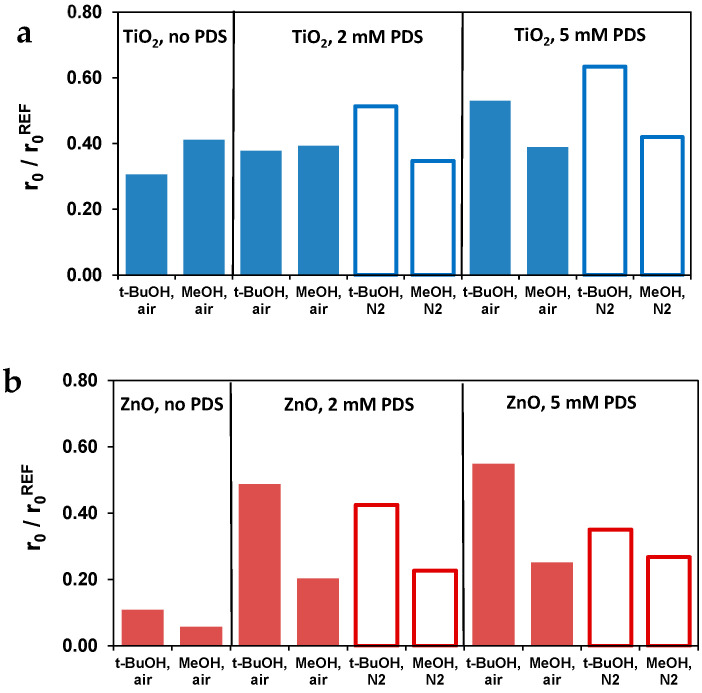
The relative initial transformation rates of TRIM in the presence of t-BuOH and MeOH at different PDS concentrations in the case of TiO_2_ (**a**) and ZnO (**b**). (r_0_^REF^: the initial transformation rate without radical scavenger; r_0_: the initial transformation rate in the presence of radical scavenger).

Without PDS, the inhibition effect of t-BuOH and MeOH confirms the significant contribution of ^•^OH-based transformation (Figure 6), especially for ZnO. Samy et al. [59] supposed the h_VB_^+^-driven TRIM transformation in TiO_2_ suspension, which can explain the lower inhibitory effect of radical scavengers (Figure 6). Using 2.0 × 10^−3^ M PD, in aerated suspension, there is no difference between the effect of t-BuOH and MeOH for TiO_2_, underlining that the ^•^OH remains the dominant reaction partner. The moderated inhibition effect of t-BuOH can be observed in O_2_-free suspension and at higher (5 mM) PDS concentration. 

For ZnO, the effect of MeOH exceeds that of t-BuOH, proving the significant contribution of SO_4_^•−^ to the TRIM transformation (Figure 6). The inhibition effect of t-BuOH and MeOH is less pronounced in the presence of PDS (opposite to TiO_2_, when PDS did not change the r_0_/r_0_^REF^ value, especially in the case of MeOH), suggesting that the overall contribution of radical-based transformation probably became suppressed while other processes could also participate in the TRIM transformation. In several cases, the formation and significant contribution of ^1^O_2_ to the transformation was confirmed in PDS-containing systems [13,23,60]. ^1^O_2_ reacts with TRIM (3.2 × 10^6^ M^−1^ s^−1^ [31]), however, more slowly than ^•^OH and SO_4_^•−^.

The effect of radical scavengers is consistent with our previous results; the transformation of PDS and, consequently, the contribution of SO_4_^•−^ based reaction is more favored for ZnO than TiO_2_. The effect of t-BuOH confirms that ^•^OH plays an essential role in the presence of PDS, even in O_2_-free suspensions. Moreover, the results of the competition tests also pointed out that, in addition to radical processes, the role of ^1^O_2_ can be considerable in the case of the ZnO/PDS method.

### 3.5. Aromatic Intermediates of TRIM Transformation

Organic compounds react with SO_4_^•−^ and ^•^OH; the differences lie in the preferred reaction pathway and the reaction kinetics [61,62]. The formed products were identified based on HPLC-MS (Table 1) and compared with previously published results [59,63,64,65]. For TRIM, the ^•^OH reacts primarily by addition to the aromatic ring (TRIM-OH) or via H-abstraction with -CH_2_- bridge, while SO_4_^•−^ prefers to react by electron-transfer reactions and results in keto-trimethoprim (TRIM=O) besides hydroxylated products. In the case of TiO_2_ and ZnO, the same aromatic products were formed with and without PDS, but their concentration distribution was different (Figures S4 and S6). The main reaction pathways were demethylation (P1, P2, and P4) and hydroxylation of the 1,2,3-trimethoxybenzene (P1, P2a, P2b, P4). Without PDS for TiO_2_, primarily P2, P3, and P4 form due to the hydroxylation and demethylation, while for ZnO, P6 was the main product; in addition to that, P4 and P8 form but P2 and P7, the dihydroxylation products were not detected (Figures S4 and S6). The difference reflects the distinct contribution of ^•^OH-based reaction and direct charge transfer. The PDS did not significantly change the product distribution (Figures S4–S7); therefore, reactions with SO_4_^•−^ most likely result in similar products like ^•^OH.

### 3.6. Matrix Effect

**Figure 7:**
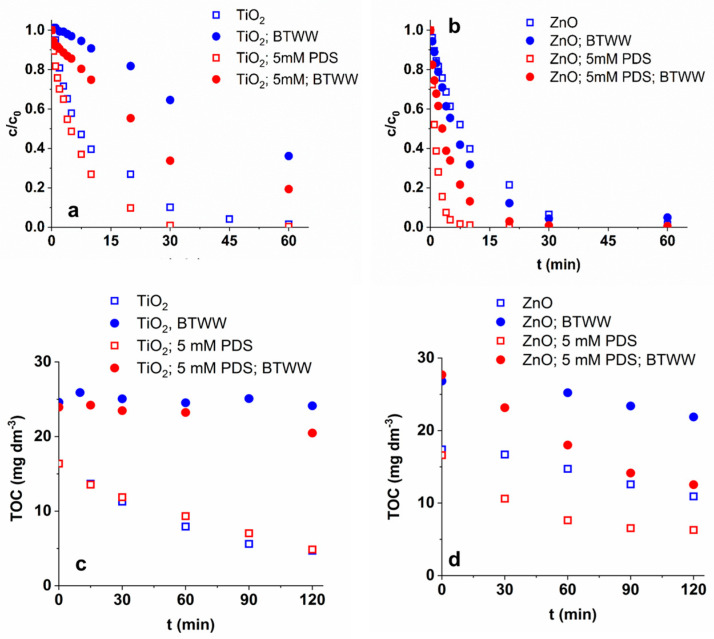
The effect of BTWW on the transformation (**a**,**b**) and mineralization (**c**,**d**) efficiency without and with PDS, in the case of TiO_2_ (**a**,**c**) and ZnO (**b**,**d**).

Experiments were performed in biologically treated domestic wastewater (BTWW) as a mild matrix (Figure 7) having a relatively low TOC value but high Cl^−^ and HCO_3_^−^ content (Table S2). Both organic substances and inorganic ions can act as radical scavengers, affecting the TRIM transformation rate via competition for reactive species. The changes in the surface properties of the photocatalyst due to the presence of inorganic ions can affect the efficiency of charge separation and radical formation [66,67,68,69,70,71]. In addition, the contribution of secondary radicals formed from inorganic ions (^•^Cl and CO_3_^•−^) must also be considered. The matrix effect was investigated without and in the presence of PDS.

In the case of TiO_2,_ the TRIM transformation and mineralization rate was significantly reduced in BTWW both with and without PDS (Figure 7 and Table 2). Using ZnO, the matrix practically does not affect the transformation and slightly decreases the mineralization rate without PDS (Figure 7b). The different inhibitory effect of BTWW suggests that the reason is most likely not the organic content of the matrix and its reaction with ^•^OH but the presence of inorganic components and their different interactions with the surface of TiO_2_ and ZnO. All this impacts the processes taking place on the surface of the photocatalysts and consequently determines the formation of radicals, the overall efficiency of the catalyst, and the transformation of organic substances.

For ZnO, in the presence of PDS, the transformation and mineralization of TRIM is most probably initiated primarily by SO_4_^•−^. As a result of the matrix, the transformation and mineralization rate decreased in this case as well, but its extent was much smaller than in the case of TiO_2_ (Figure 7 and Table 2). Comparing the initial transformation rates measured in Milli-Q and matrix, in the case of TiO_2_, it was reduced to 1.6 × 10^−8^ M s^−1^ without PDS, while to 4.0 × 10^−8^ M s^−1^ in the presence of PDS. Similarly, these values for ZnO are 1.7 × 10^−7^ M s^−1^ without PDS and 3.33 × 10^−7^ M s^−1^ in the presence of PDS. The latter is more than two times higher than the TRIM transformation rate in Milli-Q without PDS (1.58 × 10^−7^ M s^−1^) (Table 2). Although the matrix effect cannot be eliminated, the positive impact of PDS in the case of ZnO compensates for it and can even overcompensate. It is noteworthy, however, that in the presence of PDS, the negative impact of inorganic ions and the matrix is more pronounced. Without PDS, HCO_3_^−^ reduces the transformation rate by only 10%, while in the presence of PDS, it decreases it by nearly 40%. Similarly, BTWW results in a reduction only in the presence of PDS. A plausible explanation for this is the change in the radical set; due to the longer half-life of SO_4_^•−^ (30–40 µs in comparison to 20 ns of^•^OH [9,10]), the TRIM transformation is more likely to occur in the aqueous phase than on the ZnO surface. This can have consequences, such as an increased manifestation of the radical scavenging effect of inorganic ions.

The effect of the two most abundant anions, Cl^−^ (120 mg dm^−3^) and HCO_3_^−^ (525 mg dm^−3^), was investigated using the same concentrations as in BTWW (Table 2). TiO_2_ was more sensitive than ZnO to the presence of ions, especially to HCO_3_^−^ without PDS. In the presence of PDS, the inhibitory effect of inorganic ions was similar for the two catalysts (Table 2), with reference to the change in transformation processes and probably the increase in the contribution of radical-based processes.

HCO_3_^−^ may act as ^•^OH and SO_4_^•−^ scavenger and also react with h_VB_^+^. The reaction with ^•^OH and SO_4_^•−^ results in the formation of less reactive, selective carbonate radicals (CO_3_^•−^) [66]:

HCO_3_^−^ + ^•^OH → CO_3_^•−^ + H_2_O   k = 1.0 × 10^7^ M^−1^ s^−1^ (4)

HCO_3_^−^ + SO_4_^•−^ → CO_3_^•−^ + SO_4_^2−^ + H^+^  k = 2.8 × 10^6^ M^−1^ s^−1^ (5)

The reaction between HCO_3_^−^ and h_VB_^+^ also results in CO_3_^•−^ formation [66,67]:

HCO_3_^−^ + h_VB_^+^ → CO_3_^•−^ + H^+^ (6)

Using coumarin as the target substance, the impact of HCO_3_^−^ on the mineralization rate was observed in TiO_2_ suspension; this effect was moderated for ZnO [33]. Results were explained by the reaction between HCO_3_^−^ and h_VB_^+^, which is more significant in the case of TiO_2_ than in the case of ZnO. For ZnO, the HCO_3_^−^ primarily acts as a ^•^OH scavenger in the aqueous phase. TRIM reacts with CO_3_^•−^ with a lower reaction rate (1.3 × 10^7^ M^−1^ s^−1^ [72]) than ^•^OH and SO_4_^•−^, thus HCO_3_^−^ results in inhibition, especially for TiO_2_.

Cl^−^ has a much stronger radical scavenging capacity than HCO_3_^−^, as is reflected in their reaction rate constants (7)–(11). At the pH of the measurements (pH < 7), the further transformation of Cl^•^ and various reactive chlorine species (RCS) result in the reformation of ^•^OH (10) [73,74], which could be the reason that the negative impact of Cl^−^ manifests itself less than expected:

Cl^−^ + ^•^OH → Cl^•^ + OH^−^     k = 4.3 × 10^9^ M^−1^ s^−1^ (7)

Cl^•^ + Cl^−^ → Cl_2_^•−^        k = 7.8 × 10^9^ M^−1^ s^−1^ (8)

Cl^•^/Cl_2_^•−^ + H_2_O → ClOH^•−^ + H^+^ / + Cl^−^ (9)

ClOH^•−^ → ^•^OH + Cl^−^ (10)

Cl^−^ + SO_4_^•−^ → Cl^•^ + SO_4_^2−^    k = 3.6 × 10^8^ M^−1^ s^−1^ (11)

In PDS containing suspensions, the negative effect of Cl^−^ is similar to HCO_3_^−^, most probably due to the intensive decrease of SO_4_^•−^ concentration.

In the presence of both anions, the reaction between RCS and HCO_3_^−^ must be taken into consideration [73,75]:

Cl^•^ + HCO_3_^−^ → CO_3_^•−^ + Cl^−^   k = 2.8 × 10^8^ M^−1^ s^−1^ (12)

Cl_2_^•−^ + HCO_3_^−^ → CO_3_^•−^ + 2 Cl^−^ + H+   k = 8.0 × 10^7^ M^−1^ s^−1^ (13)

The products may reflect the changes in the radical set (Figures S4–S7). In the case of TiO_2_, both HCO_3_^−^ and Cl^−^ changed the product distribution, especially in the presence of PDS (Figures S4 and S5). The selectivity of CO_3_^•−^ probably caused the enhanced formation of hydroxylated products [76,77]. This effect is less pronounced for ZnO (Figures S6 and S7). Despite its negligible impact on the TRIM transformation rate, Cl^−^ changed the formation of degradation products. In the case of TiO_2_, the amount of the P4 demethylated product increased, while the hydroxylated P3 was not detected. A similar increase in P4 was observed in the presence of PDS. For ZnO, Cl^−^ impacts product distribution; the formation of P3 significantly increased, which might imply a change in the reaction mechanism, most likely due to the RCS.

### 3.7. Reusability

**Figure 8:**
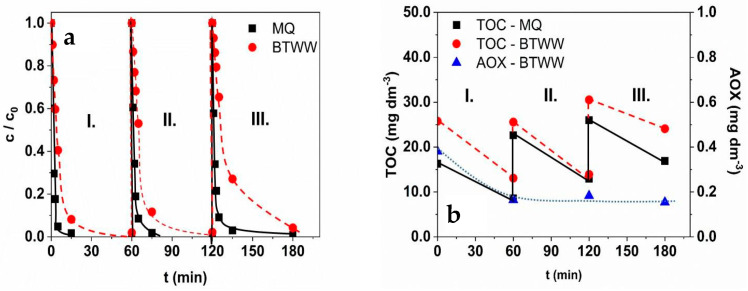
The relative concentration of TRIM (**a**) and the TOC and AOX content (**b**) over three consecutive cycles in MQ water and BTWW matrix.

From a practical point of view, it is essential to investigate the change in the activity of the catalyst and its structural stability. Due to the increased efficiency, the reusability of the ZnO photocatalysts was investigated over three runs. The catalyst was not washed between successive cycles; at the beginning of each cycle, 2.0 × 10^−3^ M PDS and 1.0 × 10^−4^ M TRIM were added to the suspension. Experiments were performed in MQ-water and BTWW matrix (Figure 8).

In Milli-Q water, the transformation rate of TRIM decreased slightly (6.02 × 10^−7^ M s^−1^ → 5.18 × 10^−7^ M s^−1^ → 5.37 × 10^−7^ M s^−1^), most probably due to the products accumulated in the treated suspension, as is demonstrated by the TOC values determined at the end of each cycle (Figure 8). 

In BTWW, the transformation was slower and reduced in each run by 15–20% (2.03 × 10^−7^ M s^−1^ → 1.72 × 10 M^−1^ s^−1^ → 1.10 × 10^−7^ M s^−1^), most likely due to the similar reason as in Milli-Q water (Figure 8). Due to the Cl^−^ content of the matrix and the possibility of RCS generation, the adsorbable organic halogen (AOX) content was measured, as reactions with RCS, especially with Cl^•^ and Cl_2_^•−^ can lead to the formation of halogenated organics. The initial AOX content of BTWW (0.4 mg dm^−3^) was reduced during the first cycle and did not exceed 0.17 mg dm^−3^ (Figure 8b). The result confirms that in the case of the PDS-combined ZnO-based heterogeneous photocatalytic process, the halogenation of organic substances is not significant even in a matrix with a high Cl^−^ content and low organic content. The decrease in catalyst activity is presumably attributable to the combined effect of the matrix and the accumulated products, which can be mitigated with appropriate treatment time and regeneration of the catalyst surface. The results of XRD and DRS measurements proved no characteristic change in the catalyst’s structure during treatments, even in the presence of PDS and inorganic ions.

## 4. Conclusions

In this work, the commercially available photocatalysts, TiO_2_ and ZnO, were combined with the peroxydisulfate ion (PDS) to enhance the charge separation and generate sulfate radical ion (SO_4_^•−^). Trimethoprim antibiotic was used as the target substance to investigate the PDS-assisted heterogeneous photocatalysis. Significant differences were found between the efficiency of ZnO and TiO_2_ for PDS activation and, consequently, for trimethoprim transformation. For ZnO, in both aerated and O_2_-free suspensions, the PDS significantly increased the transformation rate; however, in the case of TiO_2_, the positive effect was manifested in O_2_-free suspensions. The impact of dissolved O_2_ and trimethoprim on PDS transformation suggested that the interaction of individual materials with the photocatalyst surface and each other is necessary to interpret the processes on the surface and their effect on the overall efficiency. The impact of radical scavengers confirmed that ^•^OH plays an essential role in the presence of PDS even in O_2_-free suspensions, while the contribution of SO_4_^•−^ to the transformation is much more significant for ZnO than for TiO_2_. 

The biologically treated domestic wastewater was used to investigate the matrix effect. TiO_2_ was very sensitive to the impact of the matrix and its HCO_3_^−^ content; both the transformation and mineralization rate of TRIM were inhibited, while for ZnO, this effect was moderated. Most probably, the inhibition is attributed to the radical scavenging capacity of inorganic components, such as Cl^−^ and HCO_3_^−^, and their effect on the surface properties of the photocatalysts. The PDS cannot eliminate the impact of the matrix, but its positive effect on the transformation rate of TRIM can compensate for and even exceed that in the case of ZnO. 

## Figures and Tables

**Table 1:** The *m*/*z* value and structure of aromatic products of TRIM.

Name	TRIM	P1	P2a	P2b	P3
Structure	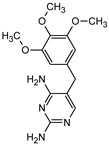	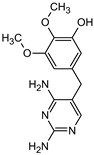	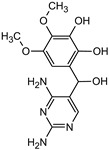	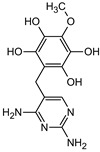	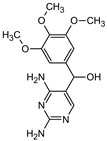
t_ret_ (min)	9.5	2.5	3.1	3.1	3.5
*m*/*z* (AMU)	291.2	277.2	309.2	295.2	307.2
Name	P4	P5	P6	P7	P8
Structure	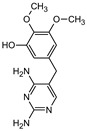		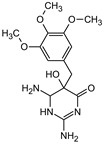	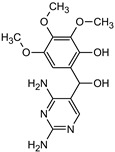	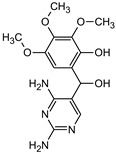
t_ret_ (min)	3.7	5.1	5.6	6.9	7.3
*m*/*z* (AMU)	277.2	only DAD detection	325.3	323.2	307.2

**Table 2:** The initial transformation rate of TRIM in MQ-water, in the presence of Cl^−^ (120 mg dm^−3^) HCO_3_^−^ (525 mg dm^−3^), and in BTWW (c_PDS_ = 5.0 × 10^−3^ M).

	r_0_ (×10^−7^ M s^−1^)			
	TiO_2_	TiO_2_/PDS	ZnO	ZnO/PDS
MQ	1.72	1.95	1.58	6.83
Cl^−^	1.43	1.40	1.56	5.18
HCO_3_^−^	1.03	1.27	1.39	4.08
Cl^−^ + HCO_3_^−^	1.05	1.25	1.45	4.72
BTWW	0.16	0.40	1.70	3.33
	r_0_^BTWW^/r_0_^MQ^			
	0.06	0.21	1.08	0.49

## Data Availability

The data is included in the article or Supplementary Materials.

## Supplementary Materials

The following supporting information can be downloaded at: https://www.mdpi.com/article/10.3390/ma16175920/s1, Table S1: The list of the chemicals, their manufacturer/distributors, and purity. Table S2: The measured parameters of the biologically treated domestic wastewater (BTWW). Figure S1: Tauc-plot analysis for determination of the band-gap value of TiO_2_ and ZnO photocatalysts. Figure S2: The relative concentration of TRIM during 365 nm photolysis, in the presence of 5.0 × 10^−3^ M PDS in the dark and under 365 nm irradiation (without catalyst), and the effect of 1.0 × 10^−2^ M SO_4_^2−^. Figure S3: The initial reaction rate (r_0_) of TRIM as a function of suspension concentration. Table S3: The initial formation rate (r_0_) of SO_4_^2−^, and the R^2^ values of the linear fitting used for the determination r_0_ values (c0(TRIM) = 1.0 × 10^−4^ M and c_0_(PDS) = 2.0 × 10^−3^ M). Figure S4: Formation of degradation products during heterogeneous photocatalysis with TiO_2_ photocatalyst. Figure S5: Formation of degradation products during PDS-assisted heterogeneous photocatalysis with TiO_2_ photocatalyst. Figure S6: Formation of degradation products during heterogeneous photocatalysis with ZnO photocatalyst. Figure S7: Formation of degradation products during PDS-assisted heterogeneous photocatalysis with ZnO photocatalyst.
