# Supplementary material for: Comparison of TiO2 and ZnO for Heterogeneous Photocatalytic Activation of the Peroxydisulfate Ion in Trimethoprim Degradation
Source: Materials (Basel). 2023 Aug 29;16(17):5920. doi: 10.3390/ma16175920 (PMC10489049; doi:10.3390/ma16175920)
Supplement: Supplementary file 1 [file materials-16-05920-s001.zip › materials-2552801-supplementary.pdf]

materials

MDPI

# Supplementary

**Table S1.** The list of the chemicals, their manufacturer/distributors, and purity

| Chemical | Manufacturer / Distributor | Purity |
|---|---|---|
| $TiO_2$ | Acros Organics | 99.5% |
| ZnO | Sigma Aldrich | 80% |
| Trimethoprim (TRIM) | Sigma Aldrich | 98% |
| $Na_2S_2O_8$ (PDS) | VWR | 99% |
| $Na_2S_2O_3$ | Alfa Aesar | 98% |
| $Na_2SO_4$ | VWR | 99% |
| $H_2SO_4$ | Sigma Aldrich | 98% |
| NaOH | VWR | 99% |
| *tert*-butanol (*t*-BuOH) | VWR | 100% |
| NaCl | VWR | 99% |
| $NaHCO_3$ | VWR | 99% |
| $Fe_2(SO_4)_3 \times nH_2O$ | VWR | 98% |
| $K_2C_2O_4$ | Reanal | 98% |
| 1,4-phenantroline | Sigma Aldrich | 99% |
| $N_2$ | Messer Hungary | 99.995% |
| Synthetic air | Messer Hungary | industrial grade |
| $H_2O$ | Merck-Millipore | ultrapure |
| Formic acid | VWR | 100% |
| Ammonium-formate | VWR | 99% |
| Methanol | VWR | 99.8% |

**Table S2.** The measured parameters of the biologically treated domestic wastewater (BTWW)

| | |
|---|---|
| pH | 7.8 |
| Conductivity (μS $cm^{-1}$) | 1258 |
| COD (mg $dm^{-3}$) | 24.4 |
| TOC[a] (C-mg $dm^{-3}$) | 10.8 |
| TIC[b] (C-mg $dm^{-3}$) | 103.4 |
| $NH_4^+$–N (mg $dm^{-3}$) | < 0.4 |
| $NO_3^-$ (mg $dm^{-3}$) | 3.37 |
| $Cl^-$ (mg $dm^{-3}$) | 120 |

[a]Total Organic Carbon

[b]Total Inorganic Carbon (sum of $CO_3^{2-}$, $HCO_3^-$ and $CO_2$)

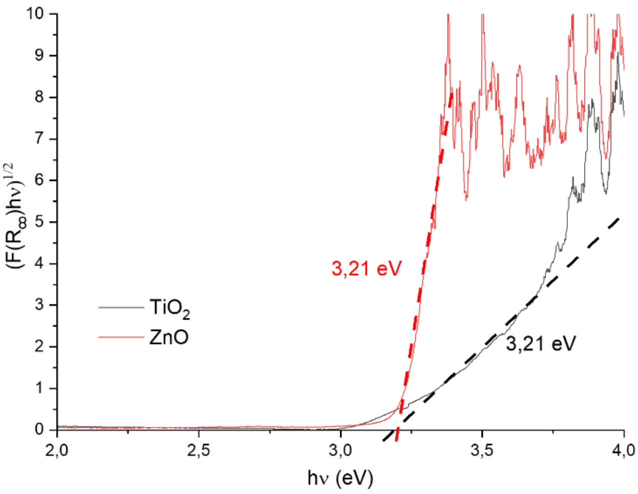


**Figure S1**. Tauc-plot analysis for determination of the band-gap value of $TiO_2$ and ZnO photocatalysts. -

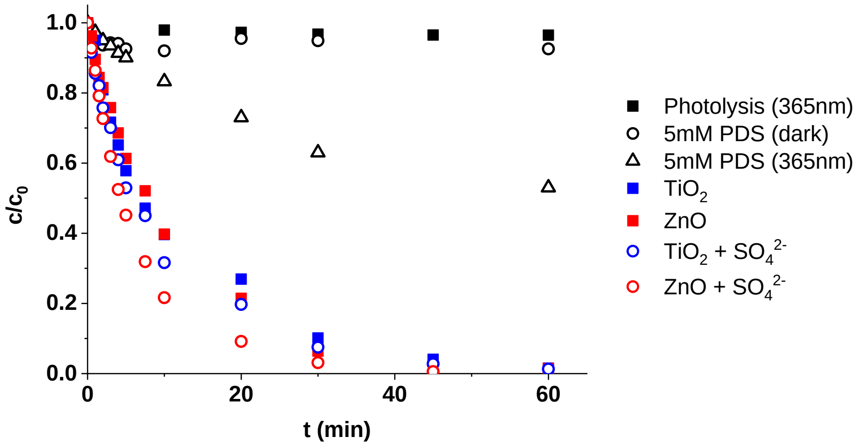


**Figure S2**. The relative concentration of TRIM during 365 nm photolysis, in the presence of 5.0×10$^{-3}$ M PDS in dark and under 365 nm irradiation (without catalyst), and the effect of 1.0×10$^{-2}$ M $SO_4^{2-}$

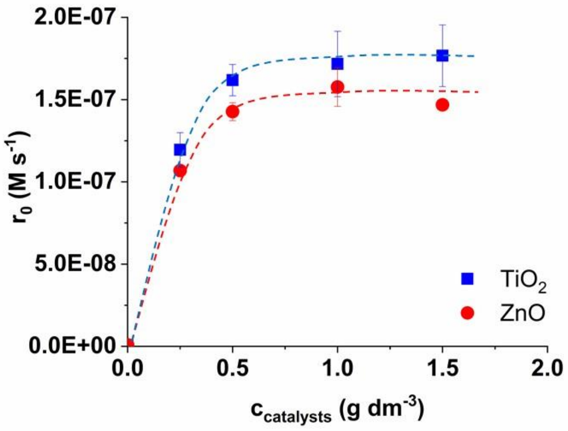


**Figure S3.** The initial reaction rate ($r_0$) of TRIM as a function of suspension concentration

**Table S3.** The initial formation rate ($r_0$) of $SO_4^{2-}$, and the $R^2$ values of the linear fitting used for the determination $r_0$ values (c0(TRIM) = 1.0×10$^{-4}$ M and $c_0$(PDS) = 2.0×10$^{-3}$ M)

| | $TiO_2$ | | ZnO | |
|---|---|---|---|---|
| | $r_0$ (M $s^{-1}$) | $R^2$ | $r_0$ (M $s^{-1}$) | $R^2$ |
| PDS/air | 2.13×10$^{-7}$ | 0.987 | 7.80×10$^{-7}$ | 0.999 |
| PDS/air/TRIM | 1.80×10$^{-7}$ | 0.991 | 1.51×10$^{-6}$ | 0.983 |
| PDS/$N_2$ | 1.65×10$^{-7}$ | 0.991 | 1.09×10$^{-6}$ | 0.999 |
| PDS/$N_2$/TRIM | 3.38×10$^{-7}$ | 0.991 | 1.74×10$^{-6}$ | 0.983 |

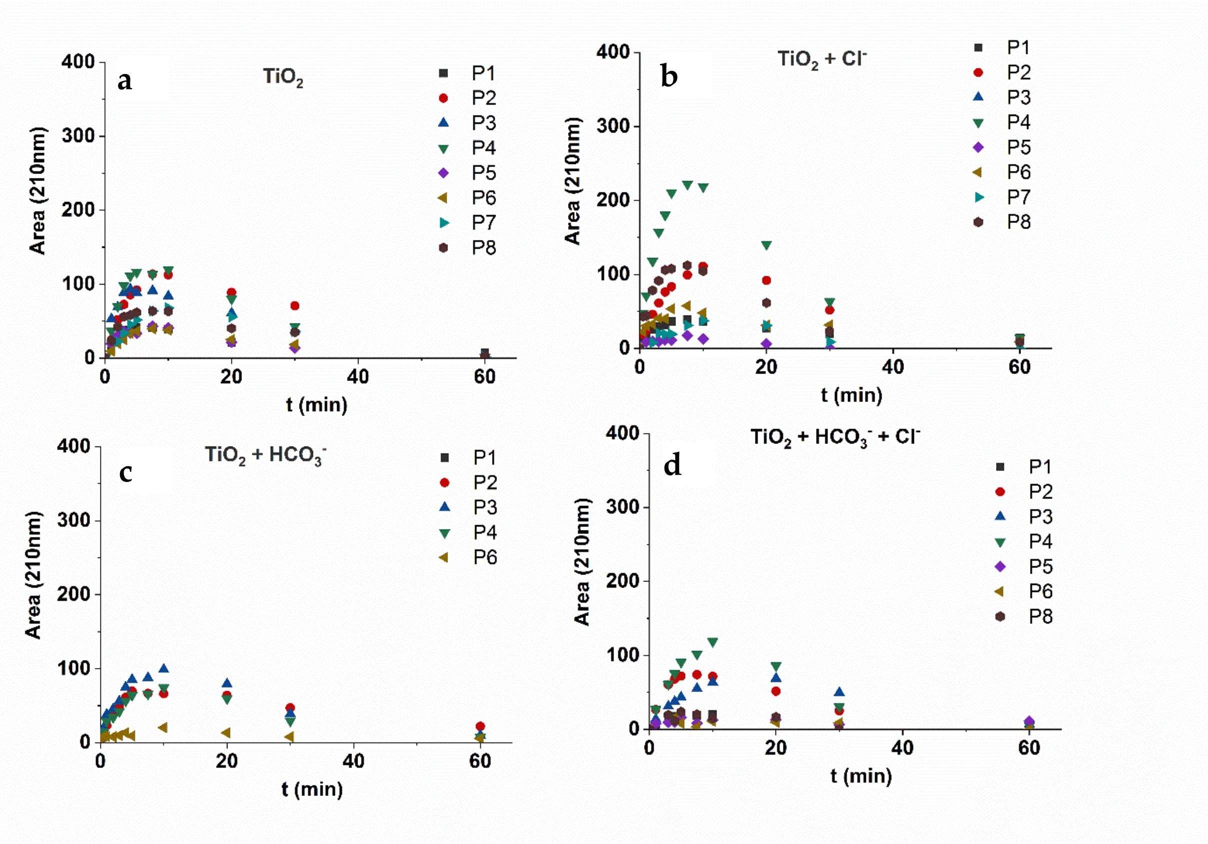


**Figure S4.** Formation of degradation products during heterogeneous photocatalysis with $TiO_2$ photocatalyst

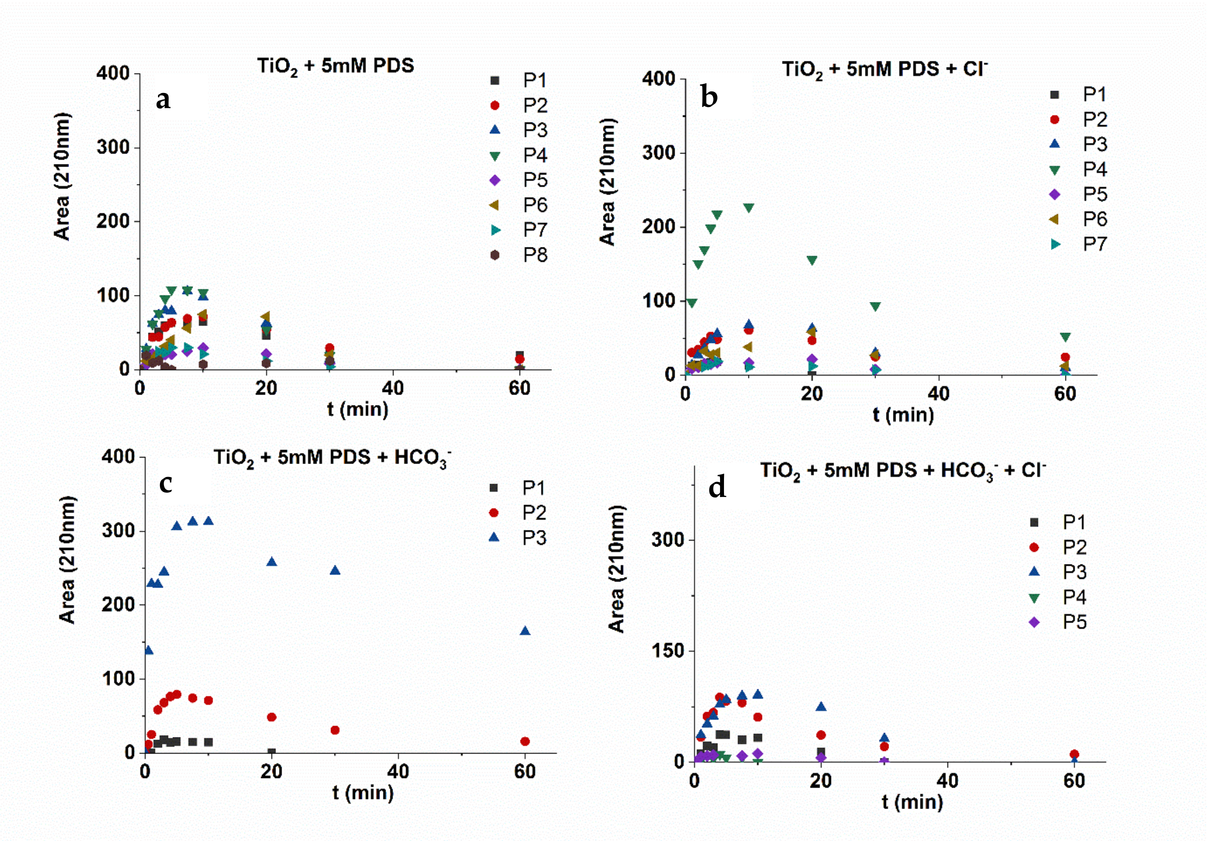


**Figure S5.** Formation of degradation products during PDS-assisted heterogeneous photocatalysis with $TiO_2$ photocatalyst

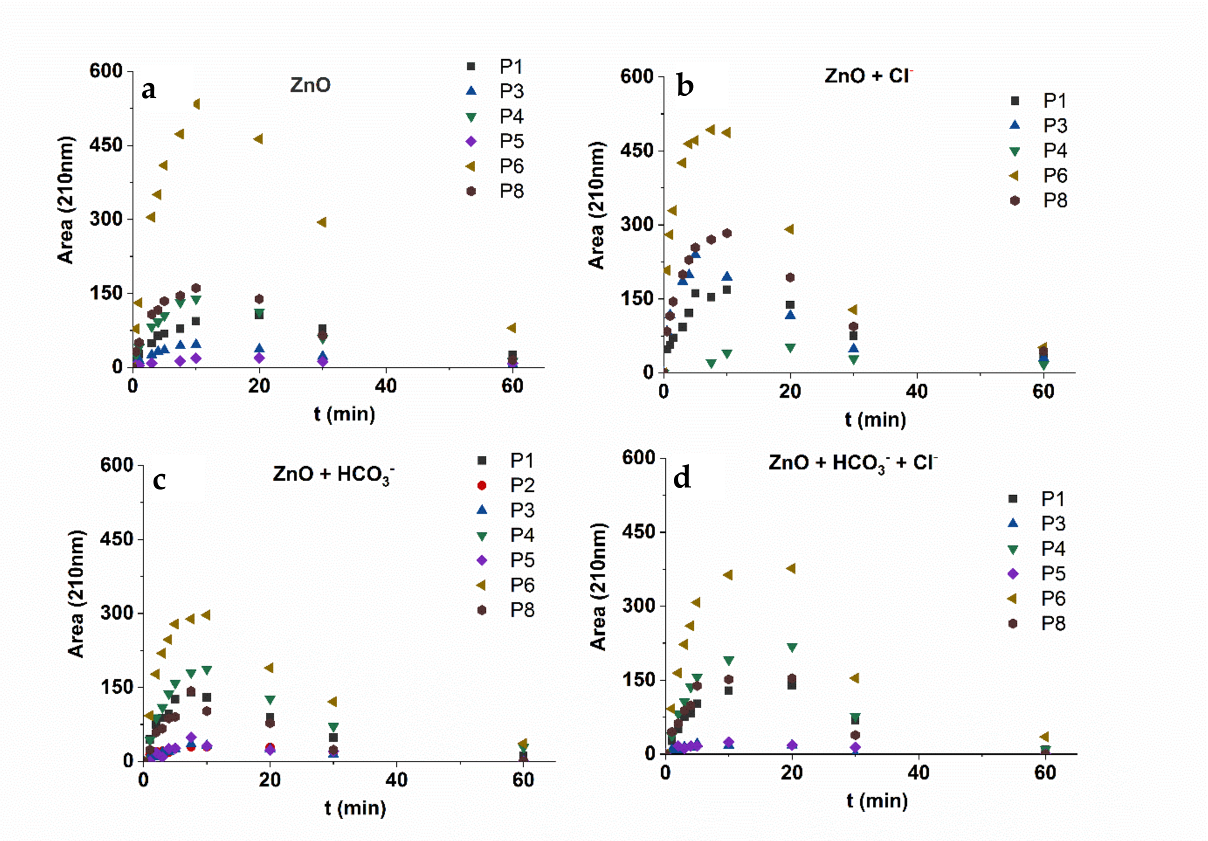


**Figure S6.** Formation of degradation products during heterogeneous photocatalysis with ZnO photocatalyst

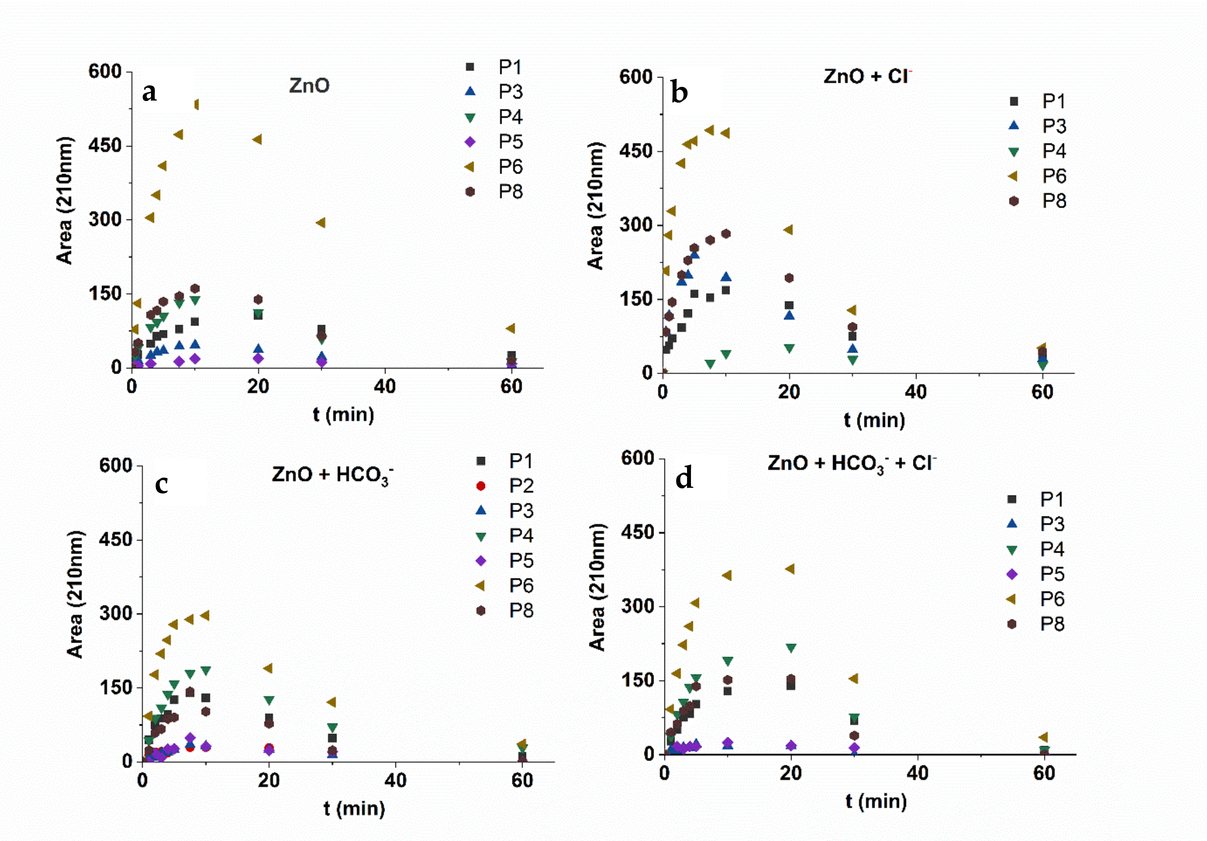


**Figure S7.** Formation of degradation products during PDS-assisted heterogeneous photocatalysis with ZnO photocatalyst
